# A 50-Year Delay in the Diagnosis of Takayasu Arteritis: Clinical Presentation and Therapeutic Insights

**DOI:** 10.7759/cureus.84361

**Published:** 2025-05-18

**Authors:** Takuya Yasuda, Noboru Hagino

**Affiliations:** 1 Department of Nephrology and Rheumatology, Nakadori General Hospital, Akita, JPN; 2 Division of Rheumatology, Teikyo University Medical Center in Chiba, Ichihara, JPN

**Keywords:** computed tomography angiography, delayed diagnosis, infliximab, latency, takayasu arteritis

## Abstract

Takayasu arteritis (TAK) is a rare inflammatory disease that affects large blood vessels, primarily targeting the aorta and its major branches. However, its etiology remains unknown. Although TAK is reported worldwide, it has a higher prevalence among women of Asian descent. Here, we report a rare case of TAK diagnosed 50 years after the onset of initial symptoms in a 76-year-old Japanese woman. The patient initially experienced numbness in her left upper extremities, followed by loss of consciousness. She had occasionally experienced difficulties with blood pressure measurements during routine medical examinations since the age of 20 years. Several months before admission, she had experienced visual darkening during physical activity and orthostatic dizziness that resolved with recumbency. Laboratory test results, including complete blood count, liver and renal function, C-reactive protein levels, and electrolyte levels, were within the normal limits. Computed tomography angiography (CTA) revealed stenosis of the bilateral subclavian arteries and the left common and internal carotid arteries, along with prominent collateral circulation, suggesting TAK. Given the substantial risk associated with corticosteroid-based therapy, we initiated treatment with aspirin and atorvastatin. After discharge, infliximab was started at 200 mg for three doses and then increased to a dose of 300 mg (5 mg/kg) every six weeks. Orthostatic symptoms markedly improved after the sixth infusion. Dizziness did not worsen during follow-up. Annual follow-up CTA and carotid ultrasonography showed no arterial stenosis progression.

This case report reveals that despite normal inflammatory marker levels, vascular stenosis of the carotid and subclavian arteries may cause dizziness that can be improved with infliximab therapy. Cases of TAK with marked progression despite normal inflammatory marker levels have been reported. Although the reversibility of vascular stenosis remains uncertain, infliximab therapy considerably improved dizziness and orthostatic symptoms. In patients with unexplained dizziness and orthostatic symptoms, large-vessel vasculitis, such as TAK, should be considered in the differential diagnosis, even in the absence of elevated inflammatory markers.

## Introduction

Takayasu arteritis (TAK) is a chronic inflammatory arteritis that primarily affects the aorta and its main branches. Inflammation of the blood vessels leads to wall thickening, fibrosis, stenosis, and thrombus formation [1]. TAK is commonly found among Asian populations, with Japan having reported the highest estimated prevalence in the past at 40 cases per million people [2]. Postural dizziness is one of the neurological features secondary to ischemia [3]. Dizziness is considered the most common neurological symptom in TAK, and vascular lesions that cause neurological symptoms frequently involve the common carotid artery, internal carotid artery, and subclavian artery [4].

Studies evaluating the effectiveness of computed tomography angiography (CTA) in diagnosing TAK have reported that CTA can visualize vessel wall changes such as thickening, calcification, and intramural thrombus, which are not visible with conventional angiography. The sensitivity and specificity of CTA in diagnosing TAK have been reported to be 95% and 100%, respectively [5].

In TAK, the systemic inflammatory response, manifested as an elevation in acute phase reactants, does not necessarily show a positive correlation with the inflammatory activity in the blood vessel walls. TAK can remain active even when erythrocyte sedimentation rate or C-reactive protein (CRP) levels are within normal ranges [1]. Two cases of TAK have been previously reported in which vascular stenosis progressed despite inflammation markers remaining normal [6]. These cases demonstrate that negative inflammatory markers cannot be used as evidence to rule out TAK.

We report a unique case of TAK in a patient who first presented in her 20s but remained undiagnosed for approximately 50 years due to the absence of elevated inflammatory markers. This case is notable because there have been no previous reports of TAK with such a prolonged latency period, making it an important contribution to the literature on this condition.

## Case presentation

A 76-year-old Japanese woman was admitted to our hospital after being emergently transported due to numbness in her left upper limb, followed by loss of consciousness. Since her early 20s, her blood pressure had often been unmeasurable during routine check-ups. During childbirth in her 20s, she experienced prolonged fever and was noted to have a weak pulse. The fever eventually subsided, and her symptoms resolved without requiring further examination. Several months prior to admission, she began experiencing episodes of blurred vision when exposed to bright environments. Around the same time, she started to exhibit recurrent episodes of lightheadedness upon standing or sitting, which improved within 30 minutes of lying down. At the time of admission, blood tests showed that her complete blood count, liver function, renal function, and electrolytes were within normal ranges, with her CRP level less than 0.3 mg/dL, indicating no significant inflammation (Table 1). 

**Table 1:** Admission Laboratory Data With Summary of Malignancy Surveillance (Endoscopy and Contrast-Enhanced CT)

Test Name	Value	Unit	Reference Range
Biochemistry tests			
Total protein	7.1	g/dL	6.7-8.3 g/dL
Albumin	4.2	g/dL	3.8-5.2 g/dL
Aspartate aminotransferase	31	U/L	10-40 U/L
Alanine aminotransferase	27	U/L	5-40 U/L
Lactate dehydrogenase	185	U/L	115-245 U/L
Alkaline phosphatase	251	U/L	38-113 U/L
Gamma-glutamyl transferase	18	U/L	0-30 U/L
Creatinine	0.71	mg/dL	0.47-0.79 mg/dL
Uric acid	5.3	mg/dL	2.5-7.0 mg/dL
Urea nitrogen	19.4	mg/dL	8.0-22.0 mg/dL
Glucose	100	mg/dL	79-109 mg/dL
Hemoglobin A1c	6	%	4.6-6.2 %
Triglycerides	70	mg/dL	50-149 mg/dL
Total cholesterol	217	mg/dL	150-219 mg/dL
High-density lipoprotein cholesterol	63	mg/dL	40-96 mg/dL
Low-Density lipoprotein cholesterol	134	mg/dL	70-139 mg/dL
Sodium	141	mEq/L	136-147 mEq/L
Potassium	4.4	mEq/L	3.6-5.0 mEq/L
Chloride	106	mEq/L	98-109 mEq/L
Calcium	9.8	mg/dL	8.5-10.2 mg/dL
Inorganic phosphorus	4	mg/dL	2.4-4.3 mg/dL
Total bilirubin	0.8	mg/dL	0.3-1.2 mg/dL
Estimated glomerular filtration rate	60.2	mL/min/1.73m²	>60 mL/min/1.73m²
Hematology tests			
White blood cell count	5,400	/µL	3,500-8,100 /µL
Red blood cell count	4.07×10⁶	/µL	3.76-5.00×10⁶ /µL
Hemoglobin	13.2	g/dL	11.3-15.2 g/dL
Platelet count	236,000	/µL	130,000-369,000 /µL
Mean corpuscular volume	96.1	fL	79-100 fL
Erythrocyte sedimentation rate	17	mm	3-15 mm
Coagulation tests			
Activated partial thromboplastin time	28.7	sec	25.0-38.0 sec
Prothrombin time international normalized ratio	0.99		0.9-1.1
Fibrinogen	363	mg/dL	150-400mg/dL
Immunology tests			
C-reactive protein	<0.3	mg/dL	<0.3 mg/dL
Hepatitis B virus surface antigen	Negative		Negative
Hepatitis B virus core antibody	Negative		Negative
Hepatitis B virus surface antibody	Negative		Negative
Hepatitis C virus antibody	Negative		Negative
Microbiology tests			
Serological test for syphilis	Negative		Negative
Treponema pallidum antibody	Negative		Negative
Interferon-gamma release assay	Negative		Negative
Malignancy surveillance			
Contrast-enhanced CT from chest to pelvis	Negative		
Upper gastrointestinal endoscopy	Negative		
Lower gastrointestinal endoscopy	Negative		

**Figure 1:**
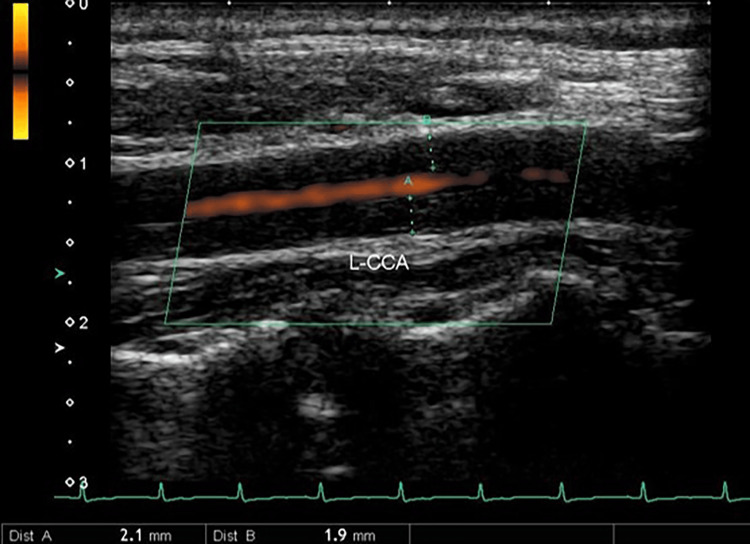
Color Doppler ultrasound image of the left carotid artery Carotid ultrasound showing circumferential thickening of the intima–media complex to approximately 2 mm in the left common carotid artery, resulting in luminal narrowing and exhibiting the “macaroni sign.”

**Figure 2:**
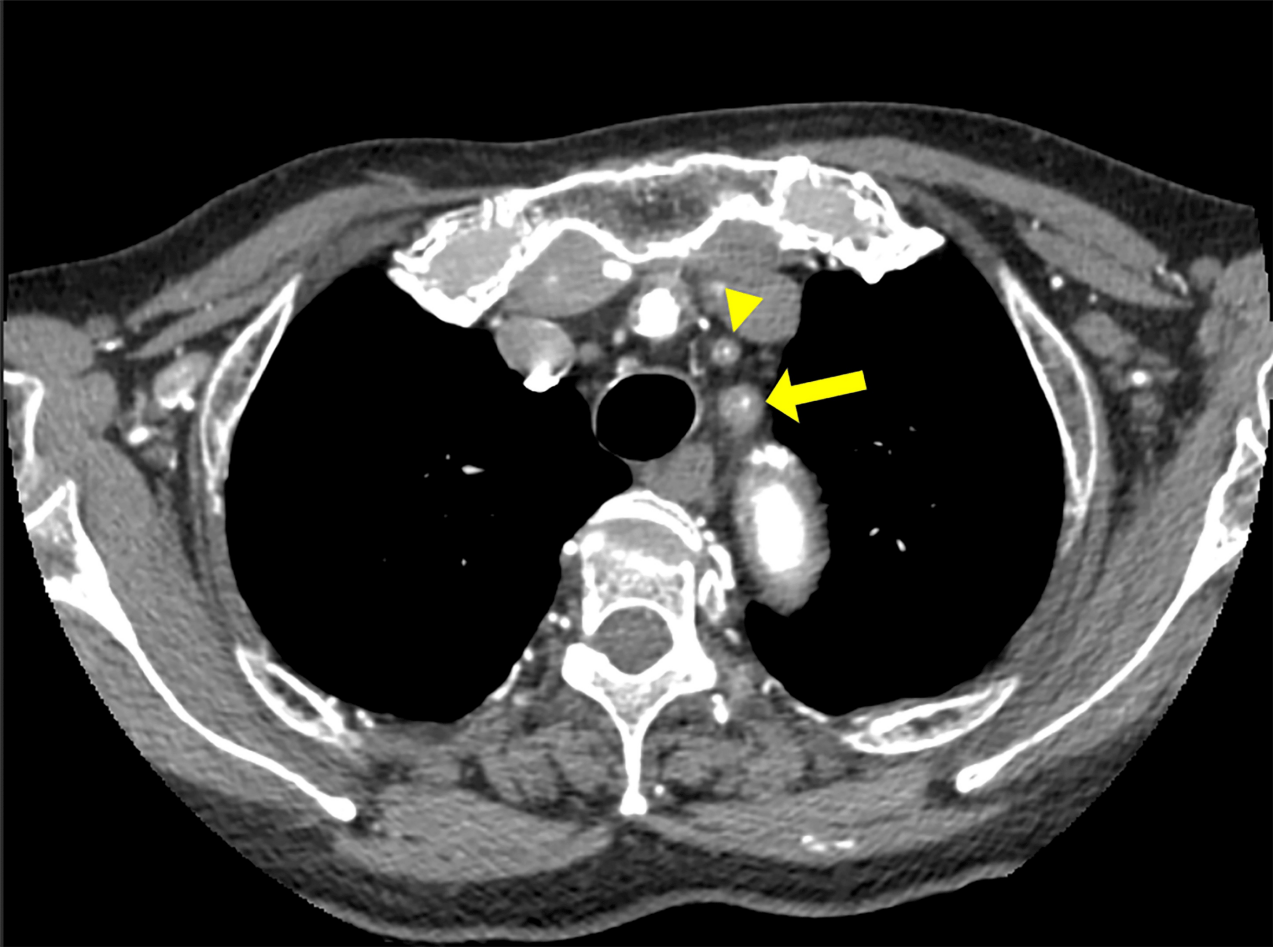
Contrast-enhanced CT axial image: Visualization of the origins of the left common carotid artery and left subclavian artery Contrast-enhanced computed tomography scan (axial view) showing severe luminal narrowing at the origins of both the left common carotid artery (arrowhead) and left subclavian artery (arrow).

**Figure 3:**
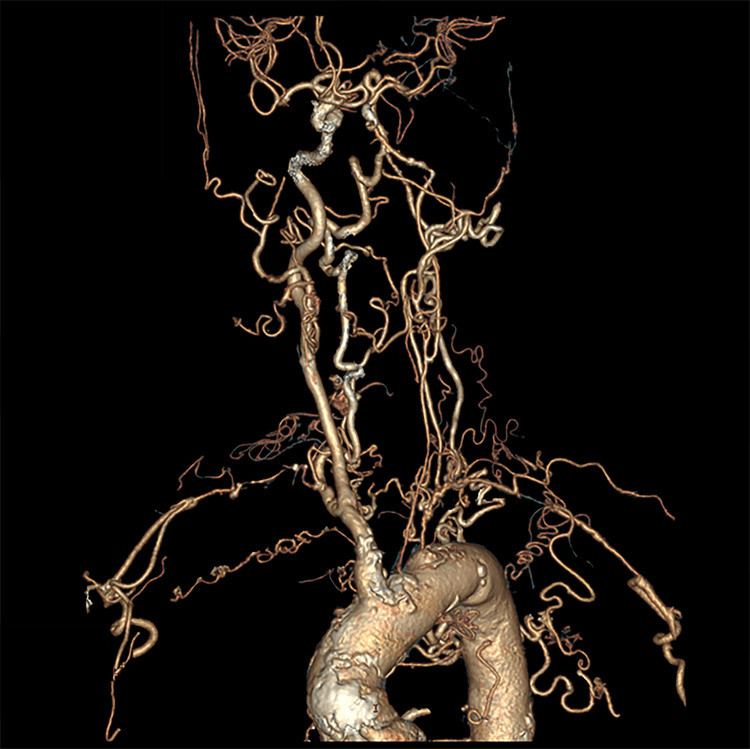
CT angiography of the aorta and craniocervical arteries Computed tomography angiography demonstrating stenosis of the left internal carotid artery, left common carotid artery, and bilateral subclavian arteries with well-developed collateral circulation.

The carotid ultrasound revealed the presence of a “macaroni sign” in the left carotid artery (Figure 1). Contrast-enhanced CT and CT angiography revealed stenosis in the left common carotid artery, left internal carotid artery, and bilateral subclavian arteries, along with considerable development of collateral circulation (Figures 2, 3). We diagnosed this patient with TAK, which had been progressing asymptomatically since her 20s. On the second hospital day, the patient was prescribed 10 mg atorvastatin, and 100 mg aspirin was added on the third day. She was discharged on the ninth day of hospitalization. Steroid-based anti-inflammatory treatment was deemed high risk and, therefore, not administered. Instead, the management plan involved administering infliximab in the outpatient setting. Before initiating infliximab therapy, we confirmed: (1) negative results for hepatitis B/C serological markers and interferon gamma release assay for tuberculosis, as shown in Table 1; (2) absence of tuberculosis family history and no tuberculosis-suggestive findings on non-contrast chest CT; (3) no malignant lesions detected by contrast-enhanced CT (chest to pelvis) and both upper and lower gastrointestinal endoscopy. Initially, 200 mg per week was administered at Weeks 2, 4, and 8 after onset. From the fourth administration (February 8, 2021), the dosage was increased to 300 mg (5 mg/kg) every six weeks. By the sixth infliximab administration, the patient reported no dizziness for a month post-treatment and noted symptom improvement; thus, the same dosage was maintained. Two years after starting infliximab, the treatment interval was extended from every six weeks to every seven weeks (300 mg) to address concerns about excessive immune suppression. Three years after initiation, the interval was further extended to every eight weeks (300 mg), and this adjustment has been maintained without worsening of dizziness. Annual follow-up CTA and carotid artery echocardiography have not shown any progression of arterial stenosis.

## Discussion

This case represents a diagnosis of TAK made 50 years after onset, triggered by the development of ischemic symptoms. TAK is a chronic large vessel vasculitis that primarily affects the aorta and its major branches, often causing progressive stenosis and occlusion [3]. As in this case, normal inflammatory markers have been reported as risk factors leading to delayed diagnosis of TAK [7]. A study of 57 TAK patients, the majority of whom were Caucasian, found that when using an ESR cutoff value of 22 mm/h, 70% of patients with elevated ESR had active TAK, while 23% had inactive TAK. Similarly, when using a CRP cutoff value of 0.6 mg/dL, 52% of patients with elevated CRP had active TAK, and 23% had inactive TAK [8]. A cohort study of 126 patients with TAK in the United States reported that the median delay in diagnosis was 17.5 months, and that the delay was even longer in patients aged 40 years or older (median delay of 44.8 months) [9], suggesting a tendency for diagnosis to be particularly delayed in older patients. The case demonstrated stenosis of the left common carotid artery, left internal carotid artery, and bilateral subclavian arteries on CTA. The patient’s clinical presentation and vascular findings supported the diagnosis despite negative inflammatory markers. This underscores the importance of imaging modalities, such as CTA, in diagnosing TAK.

Regarding treatment options for glucocorticoid- and aspirin-refractory cases, there are observational reports suggesting the effectiveness of conventional disease-modifying antirheumatic drugs (methotrexate [10], azathioprine [11], cyclophosphamide [12], mycophenolate mofetil [13], leflunomide [14]) and biologic agents (tocilizumab [15], rituximab [16], tumor necrosis factor (TNF) inhibitors: etanercept, infliximab, adalimumab, golimumab, certolizumab pegol [15]). In the case of cyclophosphamide, although partial efficacy was observed in approximately half of the patients, there were many adverse effects, mainly infections [1]. Although the treatment of TAK is controversial, TNF inhibitors, such as infliximab, have shown therapeutic potential [17]. A report indicated that the proportion of TNF-α-producing T cells is significantly higher in patients with active TAK compared with that in those in remission or healthy controls [18]. Although the reversibility of vascular stenosis remains unclear, administration of infliximab resulted in marked improvement in dizziness and orthostatic symptoms, demonstrating its efficacy in symptom management and improvement in quality of life, even in chronic cases with established vascular stenosis. Clinicians should consider TAK as a potential diagnosis in patients presenting with dizziness and orthostatic symptoms, even when the laboratory findings are inconclusive.

In patients receiving infliximab, especially in cases where treatment has been continued for more than two years, as in the present case, the addition of azathioprine has been shown to significantly reduce the formation of anti-infliximab antibodies compared to infliximab monotherapy [19]. However, in this case, the patient was 78 years old, and the interval of infliximab administration was already being extended due to concerns about excessive immunosuppression. Adding azathioprine was considered to further increase the risk of infections and adverse events, which are more common in elderly patients. Since a sufficient clinical response was achieved with infliximab monotherapy, azathioprine was not added. Routine surveillance for hepatitis B virus (HBV) reactivation was considered unnecessary, as the patient tested negative for hepatitis B surface (HBs) antigen, hepatitis B core antibody, and hepatitis B surface antibody [20]. However, to prevent excessive immunosuppression, the dosing interval of infliximab was gradually extended over the course of a year, from every six weeks to every seven weeks, and then to every eight weeks. If hepatic dysfunction or elevation of ALT suggestive of hepatitis were to develop during treatment, we planned to perform additional investigations, including HBV DNA and highly sensitive HBs antigen assays, to evaluate for HBV reactivation. Regarding malignancy surveillance, although annual upper and lower gastrointestinal endoscopy is generally recommended, we opted to substitute this with annual contrast-enhanced CT from the neck to the pelvis, performed for disease activity assessment of Takayasu arteritis, considering the patient’s advanced age and the burden of endoscopic procedures. In general, endoscopy is reserved for cases demonstrating progressive anemia.

## Conclusions

This report presents a rare case of TAK diagnosed five decades after the initial onset of symptoms. It demonstrates that in cases presenting with unexplained dizziness and orthostatic symptoms, the possibility of large vessel vasculitis such as TAK should be considered, even without inflammatory markers. Early recognition and appropriate management are essential to prevent disease progression and associated complications. Atherosclerosis may be a confounding factor in elderly-onset Takayasu arteritis and should be carefully considered when interpreting clinical and imaging findings. This case also suggests the potential long-term efficacy of infliximab as a treatment for TAK with negative inflammatory responses and progressive vascular stenosis.
